# New Insights into the Microbiota of Moth Pests

**DOI:** 10.3390/ijms18112450

**Published:** 2017-11-18

**Authors:** Valeria Mereghetti, Bessem Chouaia, Matteo Montagna

**Affiliations:** Dipartimento di Scienze Agrarie e Ambientali, Università degli Studi di Milano, 20122 Milan, Italy; valeria.mereghetti@unimi.it (V.M.); bessem.chouaia@unimi.it (B.C.)

**Keywords:** symbiosis, bacterial communities, crop pests, forest pests, Lepidoptera, next generation sequencing (NGS) technologies, diet, developmental stages

## Abstract

In recent years, next generation sequencing (NGS) technologies have helped to improve our understanding of the bacterial communities associated with insects, shedding light on their wide taxonomic and functional diversity. To date, little is known about the microbiota of lepidopterans, which includes some of the most damaging agricultural and forest pests worldwide. Studying their microbiota could help us better understand their ecology and offer insights into developing new pest control strategies. In this paper, we review the literature pertaining to the microbiota of lepidopterans with a focus on pests, and highlight potential recurrent patterns regarding microbiota structure and composition.

## 1. Introduction

Insects represent the most successful taxa of eukaryotic life, being able to colonize almost all environments, including Antarctica, which is populated by some species of chironomids (e.g., *Belgica antarctica*, *Eretmoptera murphyi,* and *Parochlus steinenii*) [1,2]. Many insects are beneficial to plants, playing important roles in seed dispersal, pollination, and plant defense (by feeding upon herbivores, for example) [3]. On the other hand, there are also damaging insects that feed on crops, forest and ornamental plants, or stored products, and, for these reasons, are they considered pests. Less than 0.5% of the known species of insects are considered pests, but the severe damage they cause results in losses of billions of dollars annually and represents a great challenge regarding food security [4,5]. Pest insects belong to various orders, such as Coleoptera (e.g., *Diabrotica virgifera*, *Sithophilus* spp., *Dendroctonus* spp.), Diptera (e.g., *Drosophila suzukii*, *Bactrocera oleae*), Hemiptera (e.g., *Psylla betulae*, *Psylla piri*, *Aphis* spp., *Myzus persicae*, *Chermes viridis*), and Lepidoptera (e.g., *Ostrinia nubilalis*, *Eupoecilia ambiguella*, *Helicoverpa zea*, *Lymantria dispar*). Within Lepidoptera, moths, comprising approximately 160,000 species, are major pests in different parts of the world [6]. The larvae are the primary stage responsible for plant or food damage, as they feed voraciously on leaves, flowers and seeds [7]. Nowadays, several techniques are used to control pest insects, such as the sterile insect technique (SIT), chemical insecticides, or biological pest control using predators and parasitoids [8,9,10]. More recently, the potential exploitation of symbionts that are associated with insects has emerged as a promising tool in insect pest management [11]. This was made possible by a better understanding of the relationship between bacterial symbionts and their insect hosts, in part due to the advent of next generation sequencing (NGS). Many studies describe the microbial communities associated with insects using classical culture-dependent approaches, but with the advent of NGS approaches, it has been possible to further improve and refine this knowledge. Indeed, recent work suggests that the microbiota plays an important role during insect developmental stages and is involved in many host activities, such as nutrition [12,13,14,15], reproduction [3], and protection against insecticides or plant secondary metabolites (e.g., terpenes, caffeine, nicotine, cocaine, isothiocyanates) [16,17,18,19,20,21]. The symbiotic relationship between bacteria and insects is very important, given the evidence that a strict association exists between some groups of insects and a core of commensal bacteria inhabiting the gut [3,22,23]. The structure of insect bacterial communities can be influenced by a multitude of factors, such as pH, host phylogeny, life stage, host environment, and diet [24,25]. These different factors are not necessarily exclusive, but recent work has suggested that the microbiota can be altered by diet and host phylogeny [24,25,26,27]. In this review, we summarize the information regarding the bacterial communities of the principal moth pests obtained mainly using NGS approaches, organizing moth pests in two main groups: forest and crop pests. Furthermore, we investigated the possible recursive bacterial core within each moth group and potential factors that may shape the structure and composition of the moth-associated microbiota.

## 2. Why Is It Important to Study Microbiota?

Culture-independent tools, such as NGS technologies, offer the chance to interrogate the high diversity of unculturable microorganisms (bacteria, fungi, and viruses) that are present in a large variety of matrices, such as soil, foods, and animals (including arthropods) [28,29,30,31]. Furthermore, these tools provide a more comprehensive view of the host’s microbial inhabitants, allowing us to answer the general question: “What types of bacteria are present, and what are they doing”? As an example, the study of the bacterial community associated with humans has become very important for human health, both for developing new therapeutic approaches and for diagnostics (e.g., the use of microbiota signatures as biomarkers of disease presence, antibacterial molecules produced by the microbiota used for therapeutic purposes, etc.) [32]. A great number of studies and discoveries that successfully applied NGS technologies to study microorganisms in humans, e.g., the Human Microbiome Project (https://commonfund.nih.gov/hmp) and MetaHIT (metahit.eu), advanced questions about the importance of the bacterial community that is associated with animals, and in particular, insects.

Insects are colonized by a multitude of microorganisms, comprising bacteria, archaea, and eukaryotes (fungi and unicellular eukaryotes). Regarding bacteria, the main focus of this review, primarily four phyla of bacteria have been found in association with insects, namely Bacteroidetes, Firmicutes, Proteobacteria, and Actinobacteria [33]. The cuticle and gut of the insect represent the two main habitats for bacteria [33]. The cuticle presents the first barrier against microorganisms (commensals or pathogens) [34]. However, symbiotic bacteria of the genus *Pseudonocardia* are present in the exocrine glands in the cuticular crypts of some species of attine ants. These symbionts are able to protect their host by secreting antibiotics against the parasitic fungi of the genus *Escovopsis* [35]. The structure of the gut is very different between disparate insect taxa and across the developmental stages; this organ, more specifically the hindgut, represents the main habitat for bacteria [33]. Most studies have focused on the bacterial communities that are associated with the gut, and several studies have discovered that a number of symbionts in different insect orders play key roles in the fitness of their hosts [36,37,38]. These symbiotic associations represent the major driving force of evolutionary innovation by conferring novel phenotypic traits on the host, allowing for the colonization of new ecological niches. 

An enhanced understanding of the bacterial community of insects is that the resident microbiota may offer new possibilities to improve integrated pest management methods targeting economically important insects. The first step consists of investigating the host-associated bacterial community, targeting symbionts of interest and then developing strategies to use symbiont(s) to control insect pests. Monitoring the symbionts inside the insect and their possible routes of transmission represents a crucial step, because the bacterial community in insects is not always stable [39,40].

A possible option could be the use of antibiotics targeting pest symbionts to decrease the host population, but such an approach may induce the appearance and spread of antibiotic resistant bacteria. Studies have focused on developing new alternatives to antibiotics, such as disrupting the cellular processes underlying vertical transmission [41] or the nutrient interaction between the insect and bacterial symbionts [42]. Such approaches might represent new alternatives for symbiont-based control strategies [33]. 

The study of the bacterial communities associated with insect/arthropods could also improve our knowledge about the presence of potential pathogens that could be transmitted to humans. As an example, Van Treuren and colleagues [43] were able to confirm the presence of *Borrelia burgdorferi*, the vector of Lyme disease, in two tick species (*Ixodes scapularis* and *Ixodes affinis*) using 454-pyrosequencing. On the other hand, Epis and colleagues [44] showed that the strain of *Asaia* harbored by *Anopheles stephensi* was different from the opportunistic human pathogen, as described by Alazeut et al. [45], allowing for the development of potential symbiont-based control strategies against malaria. The detection of pathogens in food for human consumption is very important, especially in the case of food consumed raw, such as spices, nuts, and dry meat. Montagna and Mereghetti [46,47] showed the presence of *Staphylococcus*, *Streptococcus,* and *Burkholderia* within *P. interpunctella*, three genera implicated in human and animal disease [48,49]. 

Furthermore, NGS approaches may also allow for screening insect pathogens. As a matter of fact, the analysis of the *Apis mellifera* microbiome showed that the NGS approach is useful for the detection of bacterial pathogens (*Paenibacillus larvae* and *Melisoccoccus plutonius*) that are the causative agents of American foulbrood (EFB), a quarantine disease of the larvae and pupae of the honeybee [50,51]. Interestingly, *M. plutonius* was also found in the enzootic state, suggesting the involvement of other factors in the manifestation of EFB, such as the presence of secondary invaders (e.g., *Enterococcus faecalis*) [51].

## 3. Microbiota of Moth Pests

### 3.1. Microbiota of Forest/Garden Pests

*Choristoneura fumiferana*, the eastern spruce budworm (SBW), is a major destructive moth pest in Canada, causing extensive damage to spruce and fir [52] (Table 1). The bacterial community of this moth was studied using high-throughput sequencing [53]. Landry and colleagues [53] investigated the effects of diet and origin of the populations (laboratory vs. field populations) on the bacterial gut composition; no significant differences were observed in terms of bacterial composition, but *Pseudomonas*, a member of the phylum of Proteobacteria, was the most abundant taxon in all three groups. This result is in agreement with a previous study by van Frankenhuyzen and colleagues [54], who, using a culture-dependent approach, identified this bacterium as one of the predominant taxa in a laboratory population of *C. fumiferana*. In addition to these results, it was found that the only difference between laboratory and field *C. fumiferana* samples was the presence of the genus *Bradyrhizobium* in the lab-reared populations [53]. To date, the role played by these bacteria has not been investigated.

A similar pattern in the bacterial composition was also observed in the moth *Lymantria dispar*. The gypsy moth *L. dispar* is a highly polyphagous and invasive folivore species in North America. This insect is considered a forest pest because of its ability to attack more than 300 species of deciduous trees, including oak trees, causing economic damage to the forest industries [55,56] (Table 1). An early study examined the effect of different diets and insect origin (laboratory or field populations) on the microbiota that are associated with this moth, using traditional culture-dependent and independent approaches (i.e., PCR amplification of the bacterial 16S rRNA gene and terminal restriction fragment length polymorphism analysis: T-RFLP analysis) [57]. The results showed that the bacterial communities in *L. dispar* were very simple (only 23 phylotypes), dominated by *Enterococcus* sp. and *Enterobacter* sp. [57]. Allen and colleagues [58] confirmed this trend of bacterial structure using a metagenomic approach, but they also discovered the presence of antibiotic-resistant bacteria in the midgut of the moth. In particular, they were able to detect the presence of genes of the class β-lactamases (namely LRG-1 and RamA). These genes were assigned to *Enterobacter* sp., *Pseudomonas* and *Erwinia* sp. [58]. A different bacterial community that was associated with the same moth species and dominated by Burkholderiales was detected by Mason and Raffa [59]. They used 454-pyrosequencing to investigate the impact of the external environment (diet, laboratory, and field population) on the bacterial composition of egg masses and the midgut of different developmental stages of *L. dispar* (3rd and 5th instar larvae) [59].

Sittenfeld and colleagues [60] explored bacterial communities that are associated with the gut of *Automeris zugana*, a polyphagous caterpillar, and the impact that different diets may have on these communities. In particular, they studied the microbiota that are associated with the three gut regions of last instar larvae and pupae when the insect fed on different plants. Although only a culture-dependent approach was used, a total of 19 different species (102 isolates) were identified. The composition of the gut microbiota was different, both among caterpillars feeding on different diets and among individuals of the same feeding group. This study revealed that the most frequently occurring genus was *Enterobacter* (found in 81.1% of the samples), followed by *Micrococcus* (51.5%), and *Bacillus* (33.3%). Bacterial communities changed during development from caterpillar to pupa. Further investigation by Pinto-Tomás and colleagues [61] showed that bacteria isolated from the caterpillar guts and pupae of *A. zugana* (and *Rothschilda lebeau*) belonging to the family of Enterobacteriaceae had lipolytic and chitinolytic activities. They hypothesized that these bacteria may facilitate host digestive activities within the gut. 

The sand lily *Pancratium maritimum* is a plant belonging to the family of Amaryllidaceae that contains toxic compounds, such as alkaloids. The garden pest *Brithys crini*, also called the lily borer, feeds only on this plant. In order to understand the possible presence of gut symbionts that are able to degrade these plant secondary metabolites, the bacterial composition of the midgut and hindgut of this moth was investigated by high throughput sequencing targeting the 16S rRNA gene [40]. The results obtained by Vilanova and colleagues [62] showed that the *B. crini* microbiota was dominated by Firmicutes, assigning 94–99% of the bacterial Operational Taxonomic Units (OTUs) to *Enterococcus* sp. (Enterococcaceae). Despite their lower relative abundance (1% of the total number of reads), *Pseudomonas* sp., *Bacillus* sp., *Sphingomonas* sp., *Propionibacterium* sp., *Klebsiella* sp., and *Corynebacterium* sp. were also found in *B. crini* samples. These bacteria are known to be alkaloid degraders [62]. Such functional capabilities can be very important in allowing insects to overcome plant defense mechanisms. For example, in the coffee borer beetle *Hypothenemus hampei* (the primary coffee bean pest), it was found that the gut symbiont *Pseudomonas fulva* is able to degrade the caffeine in the beans, allowing its insect host to overcome the plant’s toxicity [63].

*Thaumetopoea pityocampa* is one of the most destructive polyphagous pests of pine forests in Europe and North Africa, feeding preferably on *Pinus* spp., *Cedrus* spp. and *Larix* spp. This pest can also be responsible for serious health problems in humans (dermatitis, conjunctivitis, and anaphylaxis) [64,65]. The bacterial community of this moth was investigated by Strano and colleagues [66] using the Illumina Miseq platform. They analyzed 4th instar larvae feeding on three different host plant species: the Aleppo pine (*Pinus halepensis*), the Maritime pine (*Pinus pinaster*), and the *Laricio* pine (*Pinus nigra* subsp. *laricio*). Generally, the microbiota of *T. pityocampa* were dominated by the phylum Proteobacteria (61%), followed by Actinobacteria (19%), Bacteroidetes (15%), and Firmicutes (3%). Within Proteobacteria, the highest numbers of OTUs were assigned to Moraxellaceae, Enterobacteriaceae, and Pseudomonadaceae. Interestingly, they found a significant difference between the bacterial community of *T. pityocampa* feeding on *P. halepensis* and the other two plants, characterized by a higher amount of Actinobacteria, more specifically *Modestobacter* [66]. 

**Figure 1:**
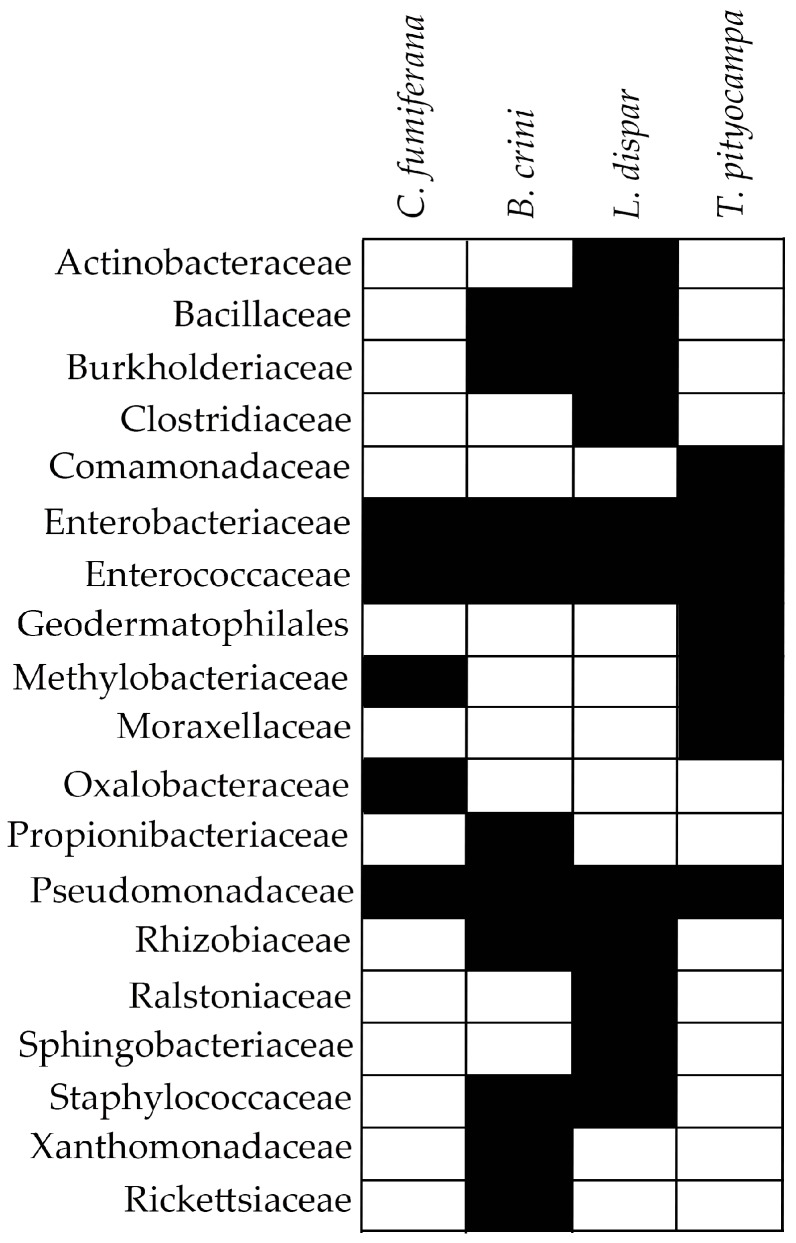
Table representing the family of bacteria shared between the microbiota of forest moth species investigated using next generation sequencing (NGS) technologies. Black-withe squares indicate the presence-absence of the bacteria family, respectively.

In the present study, a comparative analysis of the bacterial communities that are associated with the different forest moth pests studied thus far was conducted. All of the data at the level family derived from studies that used NGS technologies were used to investigate the possible presence of bacteria family/families shared across lepidopteran species. Only four species were used: *T. pytyocampa*, *L. dispar*, *B. crini,* and *C. fumiferana*. The results show that only three families were shared among the four lepidopteran species that were analyzed. These families are: Enterobacteriaceae, Enterococcaceae, and Pseudomonadaceae (Figure 1). We can hypothesize that these families were present in all of the insects investigated mainly because of their metabolic versatility and ability to help the insect overcome and degrade different complex compounds produced by plants [53,67].

### 3.2. Microbiota of Crop Pests

Moths of the genus *Spodoptera* (Table 1), also called armyworm moths, include roughly 30 species, 15 of which are recognized as agricultural pests due to their highly polyphagous activity on different crop species, causing billions of dollars of damage annually [68]. Out of the 15 known pest species, the microbiota associated with only four species have been investigated, and only *Spodoptera littoralis* (cotton leafworm) has had its bacterial community investigated using the NGS approach. The microbiota of *S. littoralis* was first investigated using classical 16S rRNA gene sequencing and microarray analysis [22]. This study showed that *Clostridium* and *Enteroccocus* (in particular *Enterococcus casseliflavus* and *Enterococcus mundtii*) represented the most abundant genera in the gut of *S. littoralis* [22]. A more recent study using in vivo pyro-SIP (coupling the pyrosequencing and stable isotope probing approaches) showed that the families Clostridiaceae, Enterococcaceae, and Enterobacteriaceae represent the functional core inside the gut of *S. littoralis* [69]. Interestingly, *E. casseliflavus* was also detected in *Spodoptera litura* [70,71] and other Lepidoptera, such as *Manduca sexta*, *Peridroma saucia*, *Bombyx mori*, *Heliothis virescens*, *Hyles euphorbiae,* and *Helicoverpa armigera* [72,73,74,75,76], and in 16 other insect taxa [77]. The association of this bacterium with a broad range of insect species suggests a possible role of *E. casseliflavus* in insects. One hypothesis is that this bacterium plays an important role in detoxification. For example, in the gut of *S. litura*, *E. casseliflavus* was able to crystallize some toxic compounds that were produced by the host plant *Phaseolus lunatus* (lima bean) (in particular α- carotenoids and β-carotenoids), forming a biofilm-like structure. This process facilitates insect adaptation to toxic compounds produced by their host plants [78]. The biofilm activity of *E. casseliflavus* was also detected in the moth *H. euphorbiae*, where investigations using scanning electron microscopy and specific PCR showed the presence of a biofilm ring at the level of the pyloric valve of the moth hindgut, suggesting a role for *E. casseliflavus* in the immobilization of toxic molecules in the plants, on which this moth feeds (*Euphorbia* sp.) [62]. From these observations, we can suggest that *E. casseliflavus* plays an important role in the protection of different Lepidoptera species against secondary metabolites contained in their host plants. 

Another dominant *Enterococcus* species in the gut of *S. littoralis* was *E. mundtii*. This bacterium was shown to become dominant starting in 6-day-old larvae of *S. littoralis* [22]. Using fluorescent in situ hybridization (FISH), it has been shown that *E. mundtii* is also able to form a biofilm-like structure inside the gut of its insect host [22,62]. Similar to the other *Enterococcus* species, *E. mundtii* was also detected in *Anticarsia gemmatalis*, *Choristoneura fumiferana*, *Mythimna separata*, and *Plutella xylostella,* where it displayed an esterase activity [53,79,80,81]. In particular, in *S. littoralis*, this species persists in the gut independent from the diet and across all of the developmental stages, suggesting an active role in the physiology of this moth [82]. On this basis, Chen and colleagues [82] suggest a clearly symbiotic relationship between *E. mundtii* and *S. littoralis*. As a matter of fact, Shao and colleagues [83] found that *E. mundtii*, present in the gut lumen, secreted an antimicrobial peptide (AMP) composed of 43 amino acid residues, called munditicin KS. This AMP was able to create an additional chemical barrier against pathogens, thereby increasing host fitness [83].

Another important agricultural pest harboring *E. casseliflavus* is the polyphagous moth *Heliothis virescens* (Tobacco budworm), a pest that is present in North and South America that is able to feed on over 80 plant species, including 19 crop species (especially clover, cantaloupe, flax, soybean, and tobacco) [5,84] (Table 1). NGS analysis of the gut microbiota of *H. virescens* revealed that the laboratory population was dominated by Enterococcaceae (73.7%), in contrast to the field populations, which harbor a more evenly proportioned and diverse bacterial community [75]. Bacterial communities of both *S. littoralis* and *H. virescens* were also investigated across different developmental stages. In both cases, the microbiota changed across the life cycle and were characterized by a simple community [75,82].

*S. litura* (Oriental leafworm moth) is another important pest of crops, feeding on cruciferous vegetables, cucurbits, groundnuts, cotton, maize, potato, soybean, and tobacco [85]. Using a culture-dependent approach, it has been discovered that, in addition to *E. casseliflavus*, a second species of the same genus is associated with this moth, namely *Enterobacter cloacae* [70]. Depending on its titer, *E. cloacae* showed insecticidal activity, causing between 30% and 70% mortality in larvae [70]. This activity has also been observed in other insects, such as *Bemisia argentifolii*, *Chrysoperla rufilabris,* and *Oberea linearis* [86,87,88]. These data suggest that this bacterium has the potential to become a promising biological control agent because of its ability to influence the immune system of *S. litura* and other insects. The presence of *E. cloacae* in this moth was also confirmed by an independent study where it was observed that, in larvae feeding on diet supplemented with the antibiotic streptomycin sulfate, *E. cloacae* disappeared and the larval mortality of *S. litura* was reduced to approximately 20% [71]. 

Using culture and enzymatic assays (filter paper enzymatic activity and β 1-4 endoglucanase activity), bacteria that are capable of enzymatically hydrolyzing lignocellulosic material were detected in the gut of *S. frugiperda* (fall armyworm), a leaf eater constituting a corn plague in Argentina [89]. In particular, similarly to other *Spodoptera* species, Firmicutes represented the most abundant phylum (62.2%), with a predominance of *Bacillus* spp., followed by Proteobacteria (24.4%), Actinobacteria (6.7%), and Bacteroidetes (6.7%) [89].

The polyphagous *Helicoverpa armigera*, also called cotton bollworm or tomato fruit borer, infests crops such as cotton, sunflower, corn, and tomato throughout the world, causing high losses in crop yields [90,91]. The microbiota of this moth was investigated in both laboratory and field populations, feeding on different diets. Independent from the diet, *Enterococcus* sp. was the dominant genus of the host-associated microbiota. These data are consistent in different studies using both culture-dependent and -independent approaches (16S rRNA sequencing and NGS approach) [22,74,76,92]. On the other hand, one study [93] using the NGS approach to investigate the microbiota of field populations of *H. armigera* feeding on tomato plants, found that the dominant bacteria belonged to the phylum of Actinobacteria, followed by Proteobacteria and Firmicutes. No reason was advanced to explain this result, but the different methodology used may be a possible cause.

The bacterial community of the diamondback moth (DBM) *Plutella xylostella* was also investigated using NGS. This insect is a worldwide pest of *Brassica* spp. crops and it has evolved a resistance to a broad spectrum of insecticides (e.g., pyrethroids, organo-phosphorous) [94,95]. Xia and colleagues [96] and Ramya and colleagues [81] both studied the bacterial communities of *P. xylostella*, with the aim of investigating the possible implications of the microbiota in insecticide resistance. Using high-throughput sequencing targeting the V6 region of the bacterial 16S rRNA, three different lines of DBM 3rd instar larvae (two lines resistant to chlorpyrifos and fipronil, respectively, and the last one susceptible to the insecticide action) were analyzed by Xia and colleagues [96]. Although, in all cases, the bacterial communities were composed of Enterobacteriales and Lactobacillales (belonging to the phylum of Proteobacteria and Firmicutes, respectively), the insecticide resistant lines exhibited a higher proportion of Lactobacillales than the susceptible strains, suggesting a role for this group of bacteria in insecticide resistance [96]. Ramya and colleagues [81] also investigated the diversity of gut bacteria of DBM larvae and adults, but studying another insecticide belonging to the oxadiazione group, indoxacarb. The results showed that *Bacillus cereus* (member of the Bacillaceae family) was able to metabolize indoxacarb, due to the presence of a carboxylesterases, and grow in the presence of this insecticide [60]. Carboxylesterase activity against this insecticide was also confirmed in other bacteria (e.g., *Alicyclobacillus tengchongensis* [97]), but in humans, it has also been found that carboxylesterases are an important mediator of drug metabolism [98]. The bacterial composition of *P. xylostella* was further investigated by Xia and colleagues [99] using a metagenomic approach. The study showed that *Enterobacter cloacae* and *Enterobacter asburiae*, members of the Proteobacteria phylum, were the dominant taxa, followed by Firmicutes. Gene enrichment followed by functional identification demonstrated that these two bacteria help their insect host by providing a series of enzymes that participate in food digestion (enzymes with a specific Brassicaceae cell wall degradation activity), nutrition (enzymes that synthetize histidine and threonine amino acids), and in the detoxification against secondary metabolites that are produced by the Brassicaceae family (particularly phenolic compounds) [99].

Similar to other agricultural pests that are analyzed using NGS techniques, the European corn borer *Ostrinia nubilalis* (ECB), where *Zea mays* is known to be the primary host, harbored a high proportion of bacteria belonging to the phylum of Firmicutes (98.5%) in a laboratory population. In contrast, field populations were dominated by Proteobacteria (62%), followed by Bacteroidetes (29%) and Firmicutes (3%) [100]. Furthermore, laboratory populations of ECB harbored several bacteria belonging to the phylum Proteobacteria (*Micrococcus* sp., *Microbacterium paraoxydans* and *Acinetobacter lwoffi*) that potentially possess enzymes for cellulose degradation [101]. The Lepidopteran *Busseola fusca* is another moth that primarily attacks maize (*Z. mays*) in Africa [102], in which the microbiota was investigated by applying culture-dependent and -independent approaches. The study carried out on larvae from 30 different maize fields [103] showed a predominance of Firmicutes (48.7%), Proteobacteria (34.6%), and Actinobacteria (16.7%), with the presence *Bacillus* and *Enterococcus* in all of th esamples, suggesting that these two genera may play a role in the fitness of *B. fusca*.

Culture-dependent techniques, in addition to DGGE (denaturing gradient gel electrophoresis) and 16S rRNA clone libraries, were used to study the bacterial community of the moth *Mythimna separata*, also called the Oriental armyworm [80]. This insect is considered a pest because of its high feeding activity on a wide range of crop plants, such as *Z. mays*, *Sorghum bicolor,* and *Oryza sativa* [104]. The results of the study showed that, of the three approaches, the 16S rRNA clone library was the most sensitive approach, identifying a high number of bacterial taxa within *M. separata*. *Escherichia* sp. represented the predominant genus (nine clones), followed by *Ralstonia pickettii* (four clones), *Ochrobactrum anthropic* (four clones), *E. mundtii* (one clone), and *Frigobacterium* sp. (one clone) [80].

*Calyptra thalictri*, also known as the fruit-piercing moth, is able to feed normally by sucking the juice of different fruits, such as raspberry, peach, plum, and citrus [105]; under particular conditions, the male of this moth becomes an opportunistic blood feeder, especially on ungulates (cattle, tapirs, and others), and, occasionally, humans [106]. The bacterial community of this moth showed the presence of bacteria belonging to the genera *Klebsiella*, *Sinorhizobium*, *Alcalignes* (*Acrhomobacter*), and *Rhizobium* in the abdomen [107], but no further investigation regarding the microbiota was carried out. 

**Figure 2:**
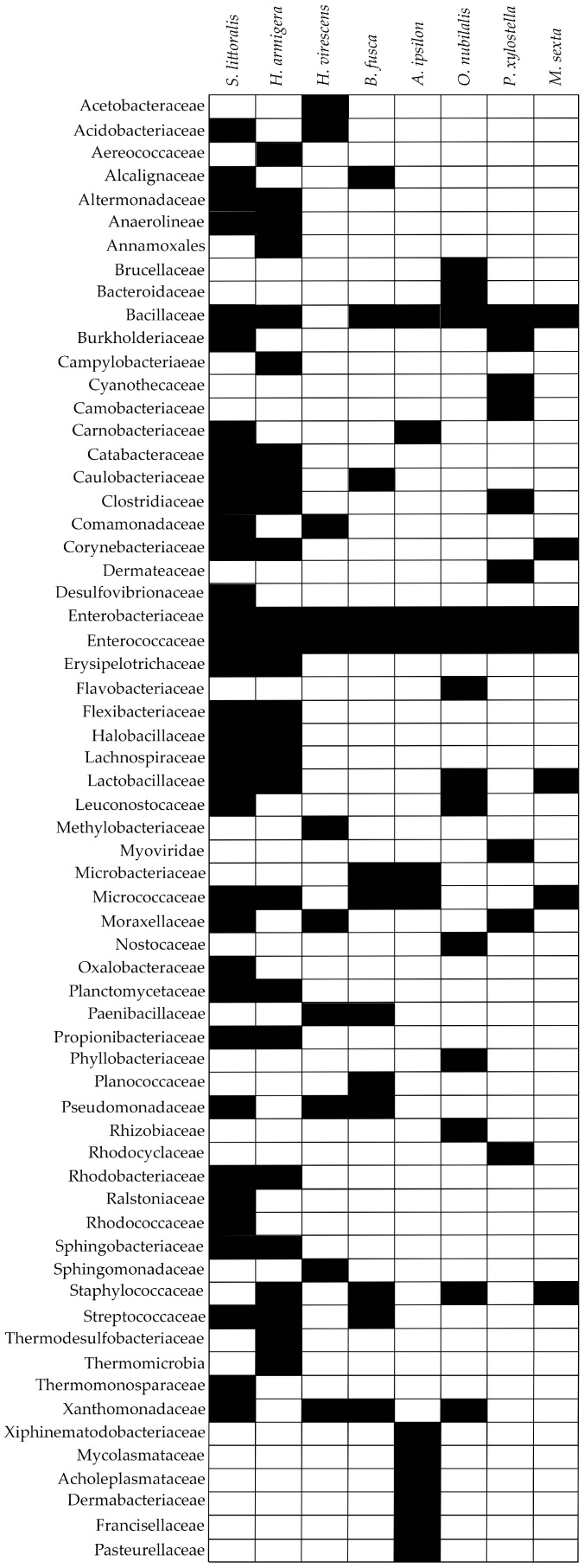
Table representing the family of bacteria shared between the microbiota of crop moth species investigated using NGS technologies. Black-withe squares indicate the presence-absence of the bacteria family, respectively.

Similar to the forest moths, a comparative analysis of the taxa harbored by agricultural moth pests was carried out. The information at the family level obtained from the NGS studies of the microbiota that are associated with these pests was summarized. In this case, a larger number of insect species was analyzed, namely: *S. littoralis*, *H. armigera*, *H. virescens*, *B. fusca*, *A. ipsilon*, *O. nubilalis*, *P. xylostella,* and *M. sexta.* The results show that, in the case of crop pests, the families of Enterobacteriaceae and Enterococcaceae were shared across the different lepidopteran species that were analyzed (Figure 2), suggesting the importance of these two families in the fitness of moth pests. 

### 3.3. Microbiota of Stored Products Pests

Stored products, such as seeds, dried tubers, and fruit are infested and damaged by a different group of pests, including arthropods (e.g., insects and mites) and vertebrates (e.g., rodents and birds) [108,109]. Within insects, five orders of insect are recognized as major pests of stored products: beetles (Coleoptera), moths (Lepidoptera), psocids or booklice (Psocoptera), bugs (Hemiptera), and wasps (Hymenoptera) [108]. Insect infestations of these products cause considerable economic losses [108]. Furthermore, these groups of insects represent a serious problem regarding human and animal health, because they are potentially vectors of human and animal pathogens [109]. Therefore, efficient control of these pests is necessary. Since some of the traditional approaches can be dangerous to human health (e.g., chemical insecticides), the manipulation of bacterial symbionts could represent a promising alternative for the control of these pests [110]. For this reason, the study of the bacterial community that is associated with stored product pests is important, but to date, the scope has been very limited and based mostly on culture-dependent approaches. The few studies that have investigated stored product pests focused on insects (e.g., in *Tribolium castaneum*, *Liposcelis bostrychophila*, *Acanthoscelides obtectus*, *Callosobruchus maculatus*, *Sitotroga cerealella*, and *Phthorimaea operculella* [111,112,113,114]) and mites (e.g., *Tyrophagus putrescentiae* [115,116]). 

Concerning moths, damage to stored products is primarily due to the feeding activity of larvae and by the produced silk webs and frass. To date, only the bacterial community of the Indian meal moth (IMM) *Plodia interpunctella* has been investigated [46,47]. *P. interpunctella* possesses a worldwide distribution and highly polyphagous activity on a wide range of stored products, such as cereals, dried fruit, and meat [86]. The authors investigated the microbiota of laboratory populations, feeding on different diets (e.g., artificial diet, *Moringa oleifera* leaves and *Vicia faba* beans), as well as that of field populations feeding on *Capsicum annuum* chili and white-black buckwheat (used to make pizzoccheri) [46]. The results showed the existence of two distinct communities, named entomotypes, based on the protein and carbohydrate content of the diet [46]. Firmicutes was the dominant component of the microbiota that is associated with moth feeding on artificial diet and pizzoccheri (both carbohydrate-rich diets), while the other groups did not show the presence of dominant taxa. Furthermore, the bacterial composition associated with the IMM was also investigated across its developmental stages [47]. In this second study, the authors adopted a different diet to that used previously and, for this reason, it was difficult to compare the results of the two studies. Nonetheless, Mereghetti and colleagues [47] showed that the bacterial community remained stable across the analyzed life cycle of *P. interpunctella* (eggs, first and last instar larvae, adult females and males). Notably, all of the developmental stages were dominated by the genus *Burkholderia* [47].

## 4. Factors Affecting the Moth Microbiota

### 4.1. Diet/Trophic Guilds

The impact of the host diet on host-associated microbiota has been demonstrated in a plethora of studies and in different insect orders, such as Diptera (e.g., *Drosophila* sp.) [117], Coleoptera (e.g., red palm weevil, *Cryptocephalus marginellus* species complex) [24,26], Hemiptera (e.g., wine mealybug *Planococcus ficus*) [118], and Blattodea (e.g., cockroach) [119,120].

Moths are globally distributed and are able to feed on different substrates, such as leaves, flowers, seeds, and human stored products [121]. Furthermore, they display a wide range of phenotypes from monophagous to highly polyphagous; such habits have been linked to their solar activity (e.g., nocturnal or diurnal species) [122].

The literature regarding moth pests available thus far seems to indicate that bacterial gut communities are greatly influenced by diet. These results have been observed using both culture-dependent and culture-independent approaches based on NGS technology [22,46,57,60,66,75,92,100]. For example, in a study investigating the effect of diet on *P. interpunctella* microbiota [46], the authors suggested that different diet compositions promote changes in the pre-existing bacterial community, describing two distinct entomotypes: (i) entomotype *Atopococcus* associated with moths that fed on an artificial diet or pizzoccheri, with a high carbohydrate content, and; (ii) entomotype *Propionibacterium* associated with moths fed on *Moringa oleifera* leaves, chili, or *Vicia faba* beans, with a high protein content. Similarly, it has been observed that the cricket microbiota differed when insects fed on a protein-rich diet when compared to when they fed on a fiber-rich diet [123]. Furthermore, if we compare the bacterial community of *P. interpunctella* with the microbiota of the mites *Tyrophagus putrescentiae* and *Carpoglyphus lactis* [115,116,124], which belong to a different group but feed on the same stored products [125,126], no common pattern regarding bacterial composition emerges. The bacterial communities associated with these two mite genera were characterized by the presence of *Leuconostoc*, *Elizabethkingia*, *Solitalea*, *Bacillus cereus* for *Carpoglyphus lactis* [124], and *Bartonella*, *Blattabacterium* and *Solitalea* for *Tyrophagus putrescentiae* [115], while the microbiota of *P. interpunctella* was characterized by *Atopococcus*, *Proprionibacterium*, *Pseudomonas* and *Burkholderia*. These results suggest that several factors (including host phylogeny or physiology) could shape bacterial communities in addition to the food source or composition. Furthermore, 42 OTUs were shared between the different adult populations of *P. interpunctella* feeding on different diets (artificial diet, *Moringa oleifera* leaves, *Capsicuum annum* chili, pizzoccheri, *Vicia faba* beans). These OTUs were assigned to the genera *Propionibacterium*, *Corynebacterium*, *Streptococcus*, *Acinetobacter,* and *Staphylococcus*. Interestingly, Sevim and colleagues [114], using a culture-dependent approach, isolated 17 *Staphylococcus* strains from four stored product pests (*Acanthoscelides obtectus*, *Callosobruchus maculatus*, *Sitotroga cerealella,* and *Phthorimaea operculella*) reared on different substrates (beans, kidney beans, corn, and potatoes, respectively). Despite the small number of studies related to the bacterial communities associated with these insects, the results from the reported studies show that the genus *Staphylococcus* seems to be commonly associated with stored product pests and could represent a possible target for developing some biocontrol strategies, since it is possible to genetically modify bacteria of this genus [127]. 

In *Thaumetopoea pityocampa*, eight OTUs of the genus *Modestobacter* were present in the microbiota of all the studied insects, but their relative abundances changed when fed on *Pinus halepensis* rather than *Pinus nigra* subsp. *laricio* and *Pinus pinaster* [66]. A possible explanation of these results was the difference in altitude at which these three different pine species were found (300–500 m a.s.l. for *P. halepensis*, and 700 m a.s.l. for *P. pinaster* and *P. nigra*) [66]. The presence of *Modestobacter* was also correlated with the main compound found in the needles, represented by myrcene for *P. halepensis* and α-pinene for *P. pinaster* and *P. nigra*. *Modestobacter* is hypothesized to play a role in *T. pityocampa* adaptation to these species of *Pinus*. Notably, this was the first report of *Modestobacter* in Lepidoptera [66].

The bacterial community of *Automeris zugana*, a forest pest, was investigated using a culture-dependent approach [60]. *A. zugana* was reared on eight different diets (*Cydista heterophylla*, *Trigonia rugosa*, *Calycophyllum candidissimum*, *Annona purpurea*, *Inga vera*, *Quercus oleoides*, *Paullinia cururu,* and *Cordia alliodora*). The results showed that diet not only influenced the bacterial community of these moths, but also had an impact on body mass. In fact, larvae feeding on four out of eight tested diets (*C. heterophylla*, *T. rugosa*, *C. candidissimum*, *A. purpurea*) grew normally when compared to the other four tested diets. The microbiota associated with moths reared on all eight diets differed from each other, but *Serratia marcescens* was the only bacterial genus present in the moths that fed on the four plants on which *A. zugana* grows normally. These results prompted further investigations in order to understand if this bacterium plays a role in the digestion of some specific/toxic compounds within these plants. Furthermore, *S. marcescens* was described in association with other lepidopteran pests, such as *Lymantria dispar*, *Helithios virescens*, *Helicorverpa zea,* and *Ostrinia nubilalis* [57,128,129,130,131], and other insects [129]. This bacterium is not commonly pathogenic to insects, but there are reports that it may display entomopathogenic activity [132].

Two studies investigated if and how the bacterial community changes when *L. dispar* fed on different diets (i.e., commercial sterilized artificial diet, *Populus tremuloides*, *Larix laricina*, *Quercus alba,* and *Salix fragilis* in [57] and *Betula papyrifera*, *P. tremuloides* and *Q. alba* in [59]). Although the bacterial community appeared to differ between the different *L. dispar* specimens feeding on the different substrates analyzed by Broderick and colleagues [57], all of the specimens in the study by Mason and Raffa [59] appeared to be dominated by Burkholderiales. A possible explanation for the different results observed by Broderick and colleagues [54] and Mason and Raffa [56] could be in the use of different approaches in the two studies (culture-dependent approach, 16S rRNA gene sequencing, and T-RFLP technique vs. 454-pyrosequencing). Notably, bacteria belonging to the genus *Acinetobacter* were present among *L. dispar* specimens feeding on *P. tremuloides* in both studies [57,59]. Further investigation revealed that this genus was acquired by *L. dispar* from the diet (*P. tremuloides*) [133], and that it is able to degrade phenolic glycosides, a secondary metabolic compound that is produced by aspen trees [134]. Interestingly, *Acinetobacter* sp. was found as the predominant bacteria in the microbiota associated with *T. pityocampa* feeding on *P. pinaster* and *P. nigra* [66], suggesting the involvement of these bacteria in the degradation of a wide spectrum of toxic compounds within the coniferous species.

Using 454-NGS technology, the bacterial community of *Choristoneura fumiferana* was investigated. Moths fed on three different diets (artificial diet, *Abies balsamea,* and *Picea mariana*) were dominated by *Pseudomonas* spp., and in particular the proportion of this genus increased in the bacterial community of moths fed on *A. balsamea* and *P. mariana* (>80%) when compared to moths feeding on an artificial diet. Interestingly, in *Bombyx mori*, it has been demonstrated that bacteria belonging to this genus are involved in the degradation of pectin, one of the components of plant cell walls [134]. 

In *Spodoptera littoralis*, differences were observed when the moths fed on an artificial diet, showing a predominance of *Enterococcus* [22,69] when compared to when these insects fed on toxic lima beans, where the main genus was *Clostridium* [22]. Studies on the effect of the diet on the microbial composition of the pest *Heliothis virescens* showed that the microbiota of different populations fed on different diets (*Gossypium hirsutum*, *Cicer arietinum* and *Nicotiana* sp.) were different, but it also showed that different populations feeding on the same diet had different microbiota, thus indicating that multiple factors help in shaping the microbial community of this pest. 

Only one study has investigated the bacterial community associated with *Helicoverpa armigera* using NGS techniques [93]; unfortunately, they did not analyze how the bacterial community changed when the moths fed on different substrates. Interestingly, Prya and colleagues [92], using T-RFLP analysis, investigated the microbiota that are associated with leaf crops and the corresponding groups of larvae feeding on them (castor, chickpea, cotton, ladyfinger, redgram, sorghum, sunflower, and tomato), discovering that the bacteria associated with the leaf phyllospere was shared with the microbiota of the different moth groups. Furthermore, a difference in the bacterial community was detected in moths collected from different localities (Bangalore, Delhi, Pachora). As reported for *T. pityocampa* [66], but also for another insect order (e.g., *Cryptocephalus* sp. [26]), the insect-associated bacterial community was influenced by the plant’s geographical location, thereby enabling the host to adapt to the environment.

In conclusion, from these studies, we can affirm that the bacterial community associated with moth pests (or insects in general) can be influenced by the diet in two different ways: (i) a direct effect, consisting in directly conveying bacteria, more or less consistently, from the substrate to the insect; (ii) an indirect effect, by shaping the pre-existing bacterial community that is associated with the moth.

**Figure 3:**
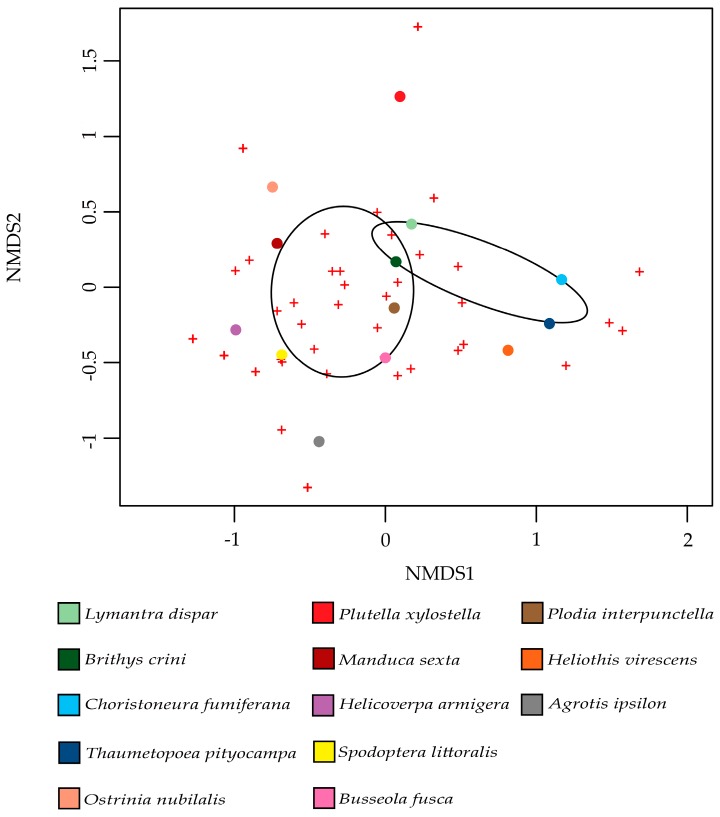
Non-metric multi-dimensional scaling analysis (NMDS) plots showing the correlation between the bacterial families associated with moths and the host plant group (angiosperms vs. gymnosperms). Circular points indicate the moths colored according to the different species, the crosses represent the bacterial Operational Taxonomic Units (OTUs) at the family level. The ellipses represent a 95% confidence area around the mean of the group.

Interestingly, a contrasting study investigating the microbial diversity associated with 31 species of butterfly, both carnivorous and herbivorous, showed that bacterial communities do not change with different diets [135]. In order to test if there was a factor influencing moth microbiota, we used the data on the moth microbiota provided by the available studies. A non-metric multi-dimensional scaling analysis (NMDS) [136] was performed and fitted with factors that are related to moth trophism that might affect the structure of the associated bacterial communities. The factors used in this analysis were: (i) feeding behavior (i.e., monophagous, oligophagous or polyphagous); (ii) the damaged plants, (gymnosperms or angiosperms); and, (iii) the type of food (leaves or seeds). The only diet factor that affected the bacterial communities (family level) of moth pests was the plant group (gymnosperms and angiosperms); *p*-value = 0.041. One group was composed of moths that feed on gymnosperms, namely *Lymantra dispar*, *Brithys crini*, *Choristoneura fumiferana,* and *Thaumatopoea pityocampa*, while the second group was composed of moth pests feeding on angiosperms: *Ostrinia nubilalis*, *Plutella xylostella*, *Manduca sexta*, *Helicoverpa armigera*, *Spodoptera littoralis*, *Busseola fusca*, *Heliothis virescens,* and *Agrotis ipsilon* (Figure 3). The fact that *Plodia interpunctella* feeds on stored products made it impossible to assign it specifically to one of these two groups, but based on the analysis, we can confirm that the bacterial community of this moth is more similar to that of moths that feed on angiosperms than to the other group (Figure 3). This result can be explained by the fact that forest moths generally feed on conifers, which contain a high variety of compounds, such as terpenes (the main component of resins in conifers) [137]. In addition, the host plants of *P. pityocampa* (*Pinus halepensis*, *Pinus nigra,* and *Pinus pinaster*) contain some specific essential oils, such as the monoterpenes myrcene and α-pinene [138], while *Pancratium maritimum*, the host plant of *B. crini*, contains potent alkaloids [139]. Monoterpenes are known to have toxic effects on insects, such as *Spodoptera litura,* or several beetle species [140,141]. As for other insects [63], we can hypothesize that the microbiota of the moths feeding on these conifers may play a role in the detoxification of such toxic compounds. Although insects have detoxification mechanisms (e.g., cytochrome P450 monooxygenases, glutathione S-transferases, and esterases) [142], some symbionts within the host-associated bacterial community are responsible for the detoxification of plant secondary metabolites. As an example, the gypsy moth *L. dispar* harbors in its microbiota some bacteria responsible for monoterpene degradation [133,134]. In other insects, such as *Dentroctonous ponderosea* (mountain pine beetle), some bacteria belonging to the genus *Pseudomonas* and *Rahnella* are able to degrade terpenes, allowing *D. ponderosea* to feed on terpene-rich trees [37]. 

### 4.2. Laboratory vs. Field Populations

In studies focused on Lepidoptera, but also on other insect orders, the general finding is that bacterial communities in laboratory reared insects are very simple and are dominated by one or a few bacterial strain(s), while communities of field populations tend to have greater diversity and evenness [76,100,143]. A clear example is represented by *Heliothis virescens*, where the microbiota of two populations, i.e., a laboratory strain fed on *Nicotiana attenuata* and a field population feeding on whole cotton (*Gossypium hirsutum*), chickpea (*Cicer arietinum*), and tobacco plants (*Nicotiana tabacum*), were compared; the results showed that no OTUs were shared between the two groups [75]. The laboratory population was dominated by Enterococcaceae, while field populations feeding on cotton, chickpea, and tobacco showed a richer microbiota composed of different bacterial families with more or less the same abundance [75]. A similar pattern was observed in the case of *Helicoverpa armigera*, *Ostrinia nubilalis,* and *Rothschildia lebeau*, where the microbiota associated with field populations were more diverse than those of laboratory populations [61,72,76,92]. A possible explanation for this trend is that, under field conditions, eggs, larvae, pupae, and adults are laid/feed on different substrates, such as the ground parts of plants, leaves, flowers, or soil, and thus are continually subjected to an enormous influx of a variety of bacterial strains. Furthermore, when field populations ingest their food, exposure to a wide range of phytochemicals or insecticides may confer more plasticity to gut microbiota, in order to help with the digestion of these substances [144]. In contrast, laboratory moths are caged in a stable environment with the same controlled artificial diet containing the same bacterial community [145]. 

Rybanska and colleagues [146] highlighted differences in four different populations of the mite *Tyrophagus putrescentiae*. They analyzed differences in both location (Bustehrad, Zvoleneves, The Netherlands, USA) and origin (laboratory and field populations) [146]. The bacterial community was different between the laboratory and the field populations, and was also associated with a difference in the growth rate between the two populations [146]. Such evidence suggests that the bacterial community can influence growth and development in moths (e.g., *A. zugana* [57]), but without clear evidence, further investigations are needed to test this hypothesis. 

This pattern of differentiation between the microbiota of field population insects and their lab-reared counterparts was not observed for *C. fumiferana,* in which populations from the field and the laboratory showed a similar bacterial composition dominated by Proteobacteria [54]. In addition, the microbiota of lab-reared populations harbored *Bradirizhobium*, which was absent from field populations. Furthermore, the number of OTUs present on the artificial diet was higher than the number of OTUs observed in the field population. For *Lymantria dispar*, despite different bacterial communities in the starting egg masses, both laboratory and field populations harbored a similarly diverse bacterial community, dominated by Burkholderiales [59]. A similar result was also observed in *Plodia interpunctella*, where different analyses confirmed that the bacterial community was not affected by the sample’s origin (laboratory vs. wild populations) [46]. 

### 4.3. Developmental Stages

Lepidoptera are holometabolous insects that are characterized by different life stages (i.e., egg, larva, pupa, and adult). The first stage is represented by eggs that hatch into larvae [81]. Larvae represent the most active stages, in which these insects feed, molt, and grow larger, until the pupal stage. Thereafter, winged adults emerge from the pupae. In general, the gut of Lepidoptera (moths and butterflies) is characterized by less compartmentalization than the gut of many other insect orders [147]. The pH is generally very alkaline, and, during metamorphosis, the gut structure changes, potentially compromising the stable colonization of the bacterial community [147]. Concerning Lepidoptera, and in particular moth pests, no clear pattern has been observed with regard to changes in microbiota structure or composition. In some insect species, developmental stages do not affect the bacterial community (e.g., *Plutella xylostella* and *Plodia interpunctella*), while in other species, bacterial communities change across the different developmental stages (e.g., *Heliothis virescens,* and *Spodoptera littoralis*). For *H. virescens*, the bacterial community changes drastically, such that no OTUs are shared across the different analyzed developmental stages [75]. In *S. littoralis*, the egg microbiota was dominated by the genus *Pantoea*, and early-instar larvae were similar to eggs, but *Pantoea* was replaced by *Enterococcus* in last-instar larvae and pupae. Furthermore, the microbiota of adults differed between genders. Males were dominated by the genus *Klebsiella*, while females showed the presence of Proteobacteria and Firmicutes in the same proportion [84]. While in *S. littoralis* the microbiota changed over development, interestingly, species of the genus *Enterococcus*, especially *Enterococcus mundtii*, were maintained during metamorphosis [84]. *Galleria mellonella* also retained *E. mundtii* throughout metamorphosis. This bacterium has been shown to play an important role in the maintenance of a “healthy” gut microbiota, conferring a protection against pathogens [148]. The bacterial community of *H. virescens* changed drastically throughout metamorphosis. 

In some species, such as *Plodia interpunctella* and *Plutella xylostellai*, the bacterial communities did not change across the analyzed developmental stages (eggs, first instar larvae, late instar larvae and adults for *P. interpunctella*; 3rd instar larvae, pupae and adults for *P. xylostella*). *Enterobacter cloacae* and *Burkholderia* sp., belonging to the phyla Proteobacteria, dominated the microbiota throughout the life cycle of *P. xylostella* and *P. interpunctella*, respectively [46,96]. The presence of *Burkholderia* with high relative abundance (average of 64%) in all of the analyzed developmental stages represents the first report describing the presence of this genus in a lepidopteran species. This bacterium has been described before in *Riptortus pedestris* and other heteropterans. In *R. pedestris*, *Burkholderia* has been found to establish a beneficial symbiosis, conferring resistance to insecticides [149,150]. The role played by this bacterium in *P. interpunctella* is still unclear, and further studies on the interaction of *P. interpunctella* and *Burkholderia* should be conducted in order to unveil its possible function.

In *Lymantria dispar*, a study on the microbial community across developmental stages (namely eggs, 3rd, and 5th instar larvae) showed that, in these different stages, Burkholderiales was the dominant taxa in the microbiota [59]. Only one study investigated the bacterial community of the butterfly *Heliconius erato* using Illumina sequencing, targeting the V4 region of the 16S rRNA gene. *H. erato*, also called the red postman, harbored a microbiota that changed across the life stages of this neotropical butterfly [151]. In particular, larvae were dominated by *Acinetobacter*, while adults showed a microbiota that was dominated by *Asaia* and *Lactococcus*. 

No signatures of vertical transmission from females to their offspring have been detected in these studies. Despite some similarity between the microbiota associated with females and eggs of *H. virescens* [72], no clear evidence supports the vertical transmission of bacteria. However, a previous study found that *Serratia marcescens* was vertically transmitted in a laboratory population of *H. virescens* [128]. Furthermore, studying the immune system of *G. mellonella*, Freitak and colleagues [152] used fluorescently-labeled *E. coli* to show that this bacterium penetrated the ovary and was subsequently found in the chorion of oviposited eggs [152]. In *P. interpunctella*, six bacterial OTUs out of 1611 were shared between eggs and adults feeding on different diets. These OTUs were assigned to *Propionibacterium* (three OTUs), *Staphylococcus* (two OTUs), and the Xanthomonadaceae family (one OTU) [46], but future studies are needed to better identify these bacteria.

The intracellular bacterium *Wolbachia* infects many arthropods [153,154]. In Lepidoptera, the vertical transmission of this bacterium has been confirmed, with an estimated incidence of infection of 84% in lepidopteran species [155]. The presence of *Wolbachia* has been found in different lepidopteran pests, such as *Colias erate*, *Ephestia cautella*, *Ephestia kuehniella*, *Ostrinia furnacalis*, *Spodoptera exempta,* and *Phyllonorycter blancardella* [156,157,158,159,160,161]. In particular, in *S. exempta*, *Wolbachia* increases the susceptibility of its host to baculovirus, making it a potential biological control agent against this crop pest [160]. 

## 5. Conclusions and Outlook

This review highlights our current knowledge on the bacterial communities that are associated with forest and agricultural moth pests, with a focus on bacterial community changes correlated with diet, developmental stage, and the origin of the population (mainly laboratory vs. field populations). It has been consistently observed that the diet and the origin of a population have a considerable impact on shaping the bacterial communities that are associated with moth pests. Regarding developmental stages, it has been found that, for some species, the bacterial community changes during the life cycle, while for others, no changes have been observed. Due to their disparity, and because relatively few studies have considered the developmental stage as a potential factor affecting the microbiota, it is hard to determine the effective impact of the developmental stage on the bacterial communities of moth pests. Furthermore, two groups of bacteria belonging to the family of Enterobacteriaceae and Enterococcaceae were present in both forest and agricultural moth pests. The advent of NGS technologies, in combination with the culture-dependent approach, will help to deepen our knowledge base regarding the microbiota of moths. In particular, NGS-based studies will improve our understanding of the ecology of the bacterial communities that are associated with these insects, helping us to understand the different factors that influence its composition, while culture-dependent approaches may help to reveal the role played by these bacteria inside their host by allowing a glimpse into their physiology with regard to host fitness. A better understanding of such interactions could be the first step in developing new ecologically-friendly symbiont-based biocontrol strategies.

## Figures and Tables

**Table 1:** Summary of the moth pests analyzed.

Moth Species	Family	Feeding Behavior	Common Host Plants	Type of Pest
*Agrotis ipsilon*	Noctuidae	polyphagous	*Zea mays*, *Gossypium* sp., *Beta vulgaris*, *Solanum lycopersicum*	crop pest
*Automeris zugana*	Saturniidae	polyphagous	*Quercus* sp., *Salix* sp.	forest pest
*Brhitys crini*	Noctuidae	monophagous	*Pancratium maritimum*	forest pest
*Busseola fusca*	Noctuidae	polyphagous	*Zea mays*, *Sorghum* sp.	crop pest
*Calyptra thalictri*	Noctuidae	polyphagous	*Citrus* sp., *Thalictrum minus*, blood from ungulates (cattle, tapirs, zebu, etc.)	crop/blood-feeding pest
*Choristoneura fumiferana*	Tortricidae	polyphagous	*Abies balsamea*, *Picea mariana*	forest pest
*Helicoverpa armigera*	Noctuidae	polyphagous	*Cucurbita pepo*, *Senecio vulgaris*, *Zea mays*, *Lycopesicon esculentum*, *Medicago sativa*, *Pisum sativum*, *Cannabis sativa*, *Hyoscyamus niger*	crop pest
*Heliothis virescens*	Noctuidae	polyphagous	*Gossypium hirsutum*, *Nicotiana tabacum*, *Cicer arietinum*	crop pest
*Lymantria dispar*	Erebidae	polyphagous	*Acer negundo*, *Acacia* sp., *Alnus alnobetula*, *Betula alleghaniensis*	forest pest
*Manduca sexta*	Sphingidae	polyphagous	*Solanum lycopersicum*, *Solanum tuberosum*, *Nicotiana tabacum*	crop pest
*Mythimna separata*	Noctuidae	polyphagous	*Avena sativa*, *Beta vulgaris*, *Brassica rapa* subsp., *Cannabis sativa*	crop pest
*Ostrinia nubilalis*	Crambidae	polyphagous	*Zea mays*, *Sorghum* sp., *Pennisetum glaucum*, *Avena sativa*, *Capsicum annuum*, *Amaranthus* sp., *Gossypium* sp.	crop pest
*Plodia interpunctella*	Pyralidae	polyphagous	Cereal grains, dried fruits, dried fish, dried meats, nuts	stored-product pest
*Plutella xylostella*	Yponomeutidae	monophagous	*Brassica* sp.	crop pest
*Rothschidia lebeau*	Saturnidae	polyphagous	*Zanthoxylum fagara*, *Fraxinus berlandieriana*, *Salix* sp., *Prunus persica*, *Citrus* sp.	crop pest
*Spodoptera frugiperda*	Noctuidae	polyphagous	*Agrostis gigantean*, *Alcea rosea*, *Allium cepa*	crop pest
*Spodoptera littoralis*	Noctuidae	polyphagous	*Beta vulgaris*, *Albemoschus esculentus*, *Asparagus officinalis*	crop pest
*Spodoptera litura*	Noctuidae	polyphagous	*Allium cepa*, *Annona squamosal*, *Arachis hypogaea*, *Beta vulgaris*	crop pest
*Thaumatopoea pytiocampa*	Thaumetopoeidae	polyphagous	*Pinus* spp., *Cedrus* spp., *Larix* spp.	forest pest
